# Radiation-induced lymphopenia correlates with survival in nasopharyngeal carcinoma: impact of treatment modality and the baseline lymphocyte count

**DOI:** 10.1186/s13014-020-01494-7

**Published:** 2020-03-14

**Authors:** Xiaoxue Xie, Shenglan Gong, Hekun Jin, Pei Yang, Ting Xu, Yilong Cai, Chengxian Guo, Rong Zhang, Fan Lou, Wenjuan Yang, Hui Wang

**Affiliations:** 1 Department of Radiation Oncology, Hunan Cancer Hospital, Affiliated Hospital of Xiangya Medical School, Central South University, Changsha, 410013 Hunan China; 2 Key Laboratory of Translational Radiation Oncology (2015TP1009), Changsha, China; 3 Radiation Oncology, The University of Texas MD Anderson Cancer Center, Houston, TX USA; 4 Center of Clinical Pharmacology, The Third Xiangya Hospital, Central South University, Changsha, China

## Abstract

**Background and purpose:**

We evaluated the relationship between patient-, tumor-, and treatment-related features and radiation-induced lymphopenia (RIL) and evaluated the correlation between RIL and survival outcome in NPC patients to help improve the treatment strategy.

**Methods:**

This retrospective study included 374 patients with stage II-IVa NPC who had been treated with definitive RT and were enrolled from 2004 to 2015; The associations between the G3–4 RIL (absolute lymphocyte count, ALC <  0.5 × 10^9^ cells/L) during RT and patient-, tumor-, and treatment-related factors were assessed using Cox regression analyses. The correlation between ALC nadir and survival was examined using a Kaplan-Meier analysis, compared with the log-rank test, and confirmed by a Cox proportional hazards analysis.

**Results:**

In the multivariate analysis, lower baseline ALC and intensity modulated radiation therapy (IMRT) (vs. 2 dimensional-conformal radiation therapy,2D-CRT) were identified as 2 independent factors that were associated with G3–4 RIL. In the multivariate survival analysis, patients with G3–4 ALC nadir had longer local recurrence-free survival durations (LRFS) (vs. G0–2 nadir, HR = 0.548, *P* = 0.005) and longer progression-free survival durations (PFS) (vs. G0–2 nadir, HR = 0.676, *P* = 0.022), while patients with G4 ALC nadir had a shorter distant-metastasis-free survival duration (DMFS) (vs. G0–2 nadir, hazard ratio [HR] = 2.567, *P* = 0.037).

**Conclusions:**

In the study, lymphopenia during RT were affected by baseline ALC and RT modality independently. Moreover, G3–4 ALC nadir was independently linked with longer PFS and LRFS durations, while G4 ALC nadir was independently linked with a shorter DMFS duration.

## Introduction

Nasopharyngeal carcinoma (NPC), which is the most common malignancy arising from the nasopharynx epithelium, is especially prevalent in southern China. Although the use of concurrent chemotherapy and intensity-modulated radiation therapy (IMRT) has provided a survival benefit [1–3], 20–30% of patients still experience tumor relapse [4]. Identifying efficient prognostic factors could help us stratify patients who are at high risk of tumor relapse.

Radiation therapy (RT) is the primary treatment for NPC. It might also directly suppress immune function via the direct destruction of mature circulating lymphocytes, a cell type that exhibits significant DNA fragmentation, even at low radiation doses (< 1 Gy) [5–7]. Tang et al. [8] found that among patients undergoing chemotherapy, higher lung V5 to V10 exhibited the greatest association with lower lymphocyte nadir (*P* <  0.001). In addition to the lungs, irradiated dose and volume of the bone marrow and spleen are also strongly associated with lymphocyte destruction [9, 10]. Therefore, it is possible that larger RT fields expose more circulating cells to radiation and thus result in lymphocyte destruction, otherwise known as radiation-induced lymphopenia (RIL).

Studies have found that absolute lymphocyte count (ALC) is a marker of immune health [11] and is correlated with patient outcomes, particularly overall survival (OS), progression-free survival (PFS), and distant metastasis-free survival (DMFS) in numerous cancers, including NPC [12, 13]. We have observed decreases in the lymphocyte population during the RT period in NPC patients. However, there is only a limited number of publications about the relationship between RIL and survival in NPC patients. Therefore, we evaluated the relationship between patient-, tumor-, and treatment-related features and RIL and evaluated the correlation between RIL and survival outcome in NPC patients.

## Materials and methods

### Patients

A total of 374 previously untreated NPC patients was enrolled between October1 2004 and May31 2015 from radiation oncology department of Hunan cancer hospital. The eligibility criteria were as follows:(1) biopsy-proven World Health Organization 1, 2, or 3, histopathologic type NPC; (2) Stage II-IVa disease according to the eighth edition of the international Union against Cancer/American Joint Committee on Cancer staging system; (3) no evidence of distant metastases before primary treatment; (4) Eastern cooperative Oncology Group performance status grade 0 or 1; and (5) A definitive radiation therapy for NPC was completed without delay.

Exclusion criteria included the following: (1) Patients without disease progression and followed less than 1 year; and (2) co-existence of a secondary malignancy, pregnancy, or lactation.

### Treatment

Of these patients, 69.8% were treated with 2D-CRT and 30.2% were treated with IMRT. 87.2% of the patients received cisplatin-based chemotherapy before or during RT; 35% of the patients received adjuvant chemotherapy. All IMRT plans and 2D-CRT plans were delivered by a 6 MV linear accelerator (Varian Medical Systems, Palo Alto, CA). Radiation was administered five times per week. All patients were treated according to the treatment principles for NPC patients at our institute.

### IMRT

The gross tumor volumes of both the primary tumor (GTVnx) and the radiologically involved cervical nodes (GTVnd) were outlined on the planning CT images with the aid of MRI images. The corresponding pGTVnx (70 Gy–74 Gy) and pGTVnd (66 Gy–68 Gy) with a 5 mm margin encompassing GTVnx and GTVnd. The clinical target volumes CTV1 (60Gy–66Gy) and CTV2 (50Gy–56Gy) respectively encompassing the high and low risk areas. The corresponding PTV1 and PTV2 with a 5 mm margin encompassing CTV1 and CTV2 were created by Boolean operations of the treatment planning system. OARs included brainstem, spinal cord, globes, optic nerves, optic chiasm, lenses, temporomandibular joints, temporal lobes auditory nerves, cochleae, mandible, oral cavity, larynx, parotid glands and vestibules. During IMRT optimization, the maximum dose of brainstem, optic nerves and chiasm must be ≤54Gy(allowing 0.1 cc brainstem <60Gy)and spinal cord ≤45Gy. Efforts were also made to limit mean dose of parotid glands to 26 Gy whenever possible and dose to the lenses and temporal lobes as low as reasonably achieved without compromising dose coverage to the PTVs.

### 2D-CRT

Two opposing lateral portals are used to cover primary nasopharyngeal cancer in nasopharyngeal cavity and adjacent normal tissue with high risk of tumor subclinical infiltration. A single front portal was used to cover the lower cervical lymph node area. 8–12 MeV electron irradiation was used to boost dose on metastatic cervical lymph nodes. Prescription dose was 70Gy–76Gy for primary tumor and 66Gy–70Gy for cervical nodes. High and low risk areas received 60Gy–66Gy and 50Gy–56Gy respectively. Brainstem must be protected by lead blocks completely when irradiation dose reach 50Gy and spinal cord must be protected by lead blocks completely when irradiation dose reach 40Gy.

### Lymphocyte count

The complete blood count was determined using a Sysmex XN-9000 automated hematology analyzer (Sysmex, Kobe, Japan). The ALC was assessed prior to RT and weekly thereafter until the completion of RT. Additional complete blood count tests were performed in patients who developed a specific condition during treatment. The ALC baseline was assessed less than 7 days before treatment (RT or induction chemotherapy), and the minimum ALC during RT was identified as the ALC nadir.

RIL of different extents was defined as grade (G) 4 (ALC <  0.2 × 10^9^ cells/L), 3 (ALC ≥0.2× 10^9^ cells/L and <  0.5 × 10^9^ cells/L), 2 (ALC ≥ 0.5 × 10^9^ cells/L and <  0.8 × 10^9^ cells/L), 1 (ALC ≥0.8 × 10^9^ cells/L and < 1.0 × 10^9^ cells/L), or 0 (ALC ≥1.0 × 10^9^ cells/L) during the RT periods (1 to 8 weeks), consistent with the lymphopenia grade (which was determined according to Common Terminology Criteria for Adverse Events [CTCAE] version 4.0). To analyze the cumulative incidence of high-level RIL (G3 and G4), we recorded the first time the ALC declined below 0.2 × 10^9^ cells/L and 0.5 × 10^9^ cells/L for each patient.

### Outcome and follow-up

The outcomes were progression-free survival (PFS), distant metastasis-free survival (DMFS), local recurrence-free survival (LRFS), and overall survival (OS) duration. The follow-up time and time to event were measured from the date of the first day of RT until the event (including recurrence and metastasis) or until the patient was censored. Patients’ medical histories were obtained, and physical examinations and nasopharyngoscopies were performed at each follow-up visit. Nasopharynx and neck MRI, chest X-ray, and abdominal sonography were routinely performed on an annual basis or upon a clinical indication of tumor relapse.

### Statistical analysis

A Cox regression model was used in the univariate and multivariate analyses to assess the effect of patient-, tumor-, and treatment-related factors on G3–4 RIL. These factors included age, sex, baseline ALC, body mass index, smoking index (pack*years), clinical disease stage, histology, chemotherapy condition, and RT modality (2D-CRT or IMRT). The criteria for including (or excluding) factors in the forward-conditional multivariate Cox regression model for high-level RIL were *P* <  0.1 for inclusion and *P* > 0.05 for removal. Kaplan-Meier 1 minus survival curves were generated for the cumulative incidence of high-level RIL by risk factors.

In the survival analysis, for each endpoint (PFS, DMFS, LRFS, and OS), outcomes by RIL based on ALC nadir values during RT were compared using the log-rank test and Cox regression analysis. Co-factors in the multivariate analysis included age, sex, baseline ALC, body mass index, smoking index (pack*years), clinical disease stage, histologic type, chemotherapy condition, and RT modality. The criteria for including (or excluding) factors in the forward-conditional multivariate Cox regression model for cumulative incidence of tumor relapse (recurrence or metastasis) were *P* <  0.1 for inclusion and *P* > 0.05 for removal.

All variables were analyzed as continuous if possible. All statistical tests were 2-sided, and analyses were performed using the SPSS ver.24.0 statistical software package (IBM Corp., Armonk, NY).

## Results

### Baseline patient characteristics

Table 1 lists the characteristics of the 374 patients (262 men and 112 women with a median age of 46 years; range, 17–70 years). The median (inter quartile range) follow-up time was 52.2 months (5.0–119.8 months). According to CTCAE 4.0, 33 (8.8%) patients had an ALC baseline < 1 × 10^9^ cells/L, and 341(91.2%) had an ALC baseline > 1 × 10^9^ cells/L.

**Table 1:** Baseline demographic, tumor, and treatment characteristics of 374 NPC patients

Characteristic	Result
Sex, N (%)	
Female	112 (29.9)
Male	262 (70.1)
Age at diagnosis, years (median, range)	46 (17–70)
ALC baseline before treatment ×10^9^ cells/L (median, range)	1.81 (0.25–3.5)
Body mass index, kg/m^2^ (median, range)	22.60 (15.60–33.77)
Smoking status, pack*year (median,range)	0 (0–120)
Tumor histologic type (WHO), N (%)	
Non-keratinized undifferentiated (III)	145 (38.8)
Non-keratinized differentiated (II)	220 (58.8)
Keratinized (I)	9 (2.41)
T status, N (%)	
T1	37 (9.9)
T2	133 (35.6)
T3	156 (41.7)
T4	48 (12.8)
N status, N (%)	
N0	31 (82.9)
N1	80 (21.4)
N2	227 (60.7)
N3	36 (9.6)
Stage^a^, N (%)	
II	46 (12.3)
III	245 (65.5)
IVa	83 (22.2)
Prescribed dose of GTVnx,Gy, (median, range)	72.7 (68–82)
Induction or concurrent chemotherapy, N (%)	
None	48 (12.8)
Induction only	62 (16.6)
Concurrent only	70 (18.7)
Both	194 (51.9)
Adjuvant chemotherapy, N (%)	
Yes	131 (35.0)
No	243 (65.0)
Radiation modality, N (%)	
2D-CRT	261 (69.8)
IMRT	113 (30.2)

*Abbreviations*: *CI* confidence interval, *HR* hazard ratio, *2D-CRT* 2-dimensional conventional radiotherapy, *IMRT* intensity modulated radiation therapy, *GTV* gross tumor volume, *WHO* World Health Organization, *ALC* absolute lymphocyte count

^a^American Joint Committee on Cancer, eighth edition

### Lymphocyte counts during RT

**Fig. 1:**
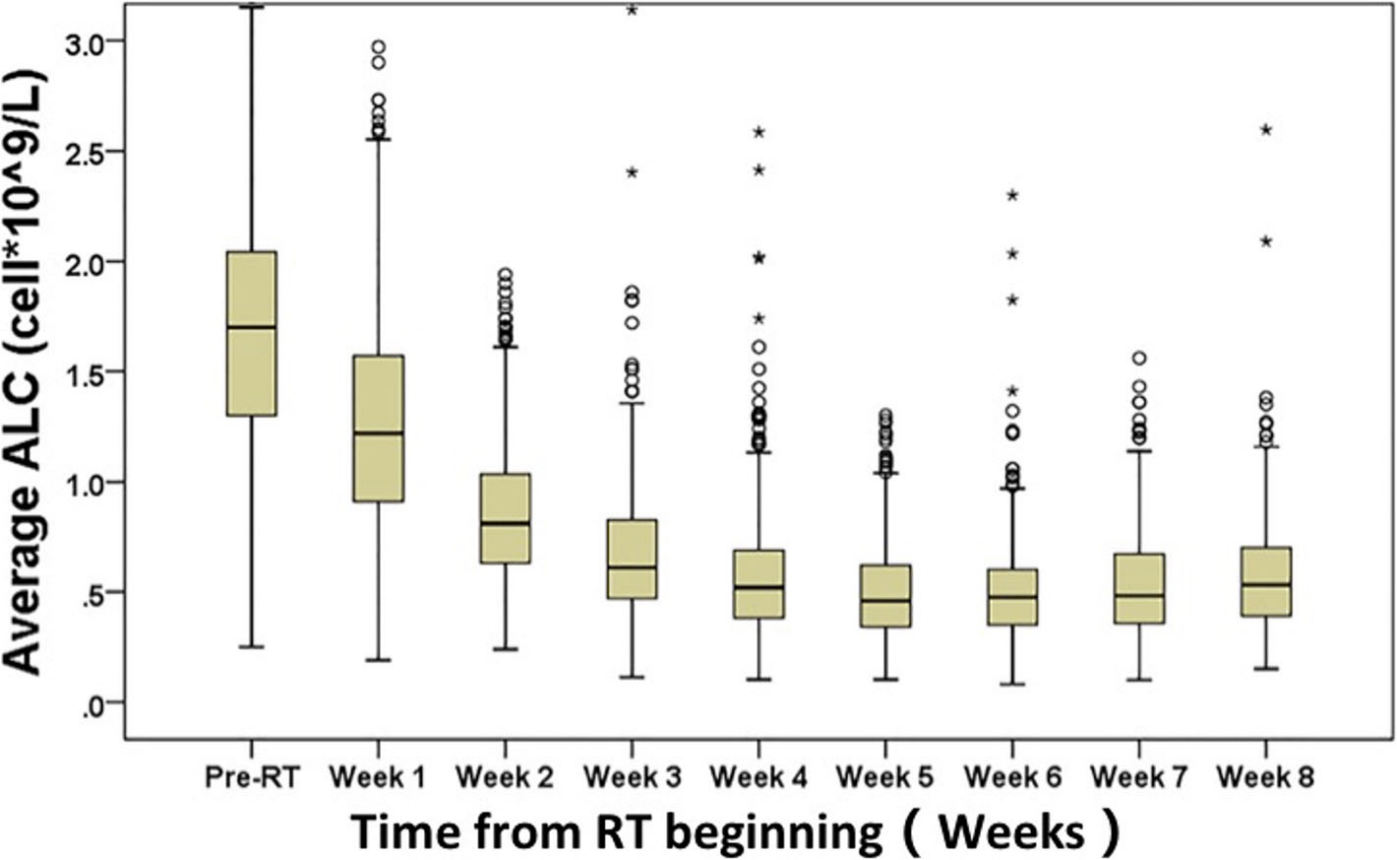
Absolute lymphocyte count (ALC) trend from before radiation therapy (pre-RT) through week 8 during RT

To visualize the peripheral blood lymphocyte trends during RT, we plotted ALCs with respect to time during RT in weeks (Fig. 1). There were 12(3.2%) patients with G0/1 nadir, 57 (15.2%) with G2 nadir, 274 (73.3%) with G3 nadir, and 31 (8.3%) with G4 nadir.

### Association between patient characteristics and incidence of high-level RIL

**Fig. 2:**
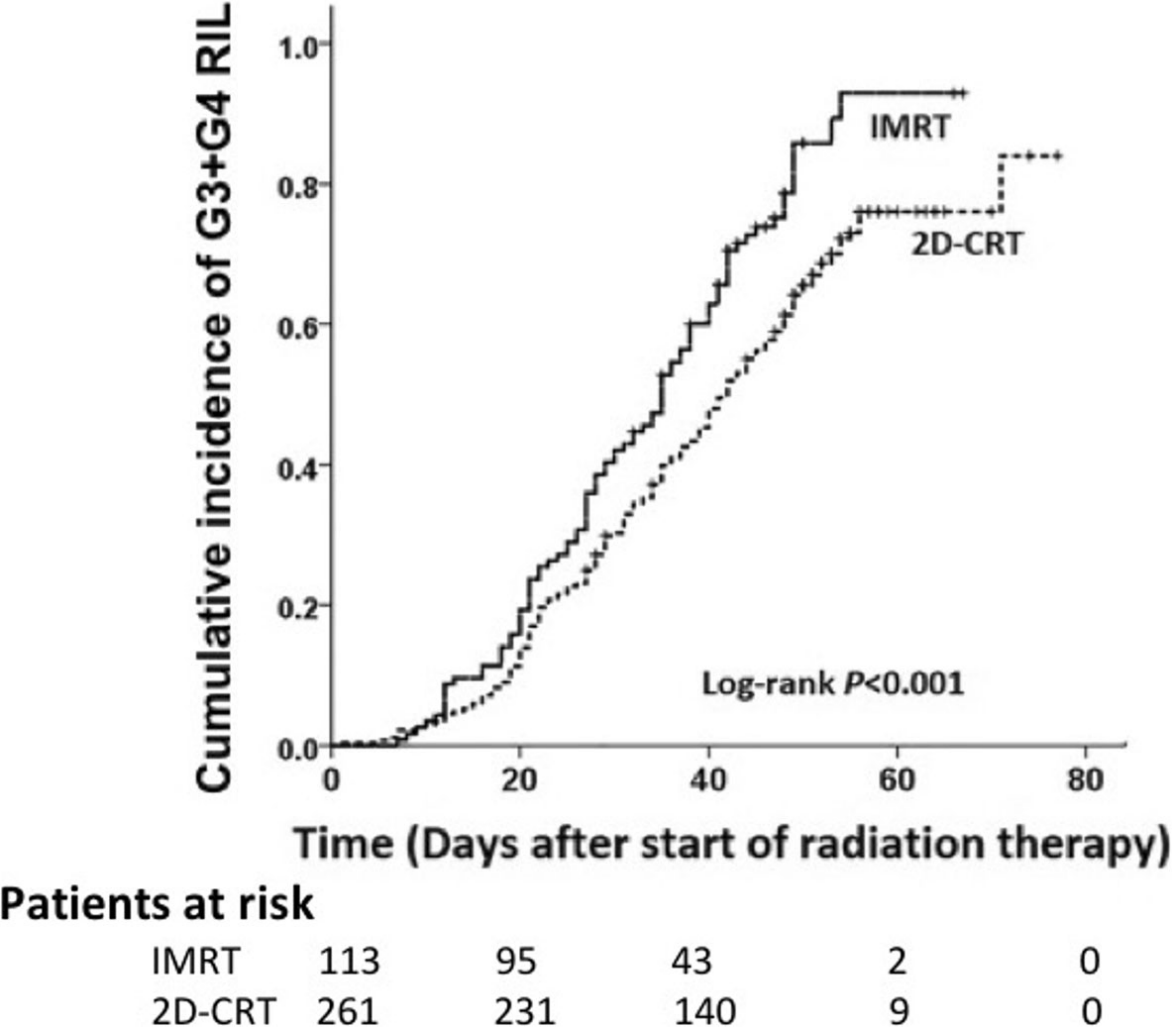
Kaplan-Meier curves show the comparison of the cumulative incidence of grade 3–4 (G3–4) radiation-induced lymphopenia (RIL) (absolute lymphocyte count [ALC] < 0.5 × 10^9^/L) after intensity-modulated radiation therapy (IMRT) and 2D-conventional radiation therapy (2D-CRT)

In the univariate analysis, we found that baseline ALC (HR = 0.591, *P* <  0.001) and RT modality (IMRT vs. 2D-CRT, HR = 1.594, *P* < 0.001) were significantly associated with the G3–4 RIL (Table 2). Cumulative incidence curves illustrate the effects of RT modality (IMRT vs. 2D-CRT) on ALC during RT (Fig. 2).

**Table 2:** Univariate Cox regression analysis demonstrates associations between baseline variables and lymphocyte nadirs during RT

Characteristic	ALC < 0.5 × 10^9^ Cells /L			ALC < 0.2 × 10^9^ Cells /L		
	HR	95% CI	*P* value	HR	95% CI	*P* value
Sex						
Female	1			1		
Male	0.997	0.772–1.288	*0.636*	1.424	0.672–3.018	*0.357*
Age at diagnosis (years)	0.992	0.979–1.004	*0.183*	0.964	0.932–0.997	***0.033***
ALC baseline	0.591	0.469–0.744	***< 0.001***	0.443	0.235–0.834	***0.012***
Body mass index	0.961	0.925–0.998	***0.041***	0.935	0.836–1.045	*0.236*
Smoking status (pack*year)	1	0.999–1.000	*0.193*	1	0.999–1.001	*0.636*
Tumor histologic type (WHO)						
Undifferentiated (III)	1			1		
Differentiated (I and II)	0.666	0.516–1.525	*0.887*	1.152	0.535–2.481	*0.717*
Stage						
II	1			1		
III	1.419	0.941–2.139	*0.102*	1.942	0.455–8.284	*0.37*
Iva	1.53	0.968–2.416	*0.097*	4.098	0.931–18.037	*0.062*
Induction or concurrent chemotherapy (N)						
None	1			1		
Induction only	1.201	0.757–1.9007	*0.971*	0.794	0.230–2.745	*0.716*
Concurrent only	1.142	0.726–1.796	*0.566*	1.221	0.409–3.644	*0.721*
Both	1.545	1.043–2.290	***0.03***	1.011	0.375–2.725	*0.983*
RT modality						
2D-CRT	1			1		
IMRT	1.594	1.233–2.061	***< 0.001***	2.148	1.099–4.200	***0.025***

*Abbreviations*: *CI* confidence interval, *HR* hazard ratio, *2D-CRT* 2-dimensional conventional radiotherapy, *IMRT* intensity modulated radiation therapy, *GTV* gross tumor volume, *WHO* World Health Organization, *ALC* absolute lymphocyte count

In the multivariate analysis, we confirmed that a lower baseline ALC and that IMRT (vs. 2D-CRT) were two independent negative factors for the G3–4 RIL (Table 3).

**Table 3:** Multivariate Cox regression analysis demonstrates two clinical characters as independent risk factors for lymphopenia during RT

Characteristic	ALC < 0.5 × 10^9^ Cells /L			ALC < 0.2 × 10^9^ Cells /L		
	HR	95% CI	*P* value	HR	95% CI	*P* value
ALC baseline	0.563	0.440–0.719	***< 0.001***	0.423	0.224–0.800	***0.008***
RT modality (IMRT vs. 2D-CRT)	1.651	1.251–2.179	***< 0.001***	2.042	1.011–4.124	***0.046***

*Abbreviations*: *ALC* absolute lymphocyte count

### Lymphocyte nadir is associated with patient outcomes

**Fig. 3:**
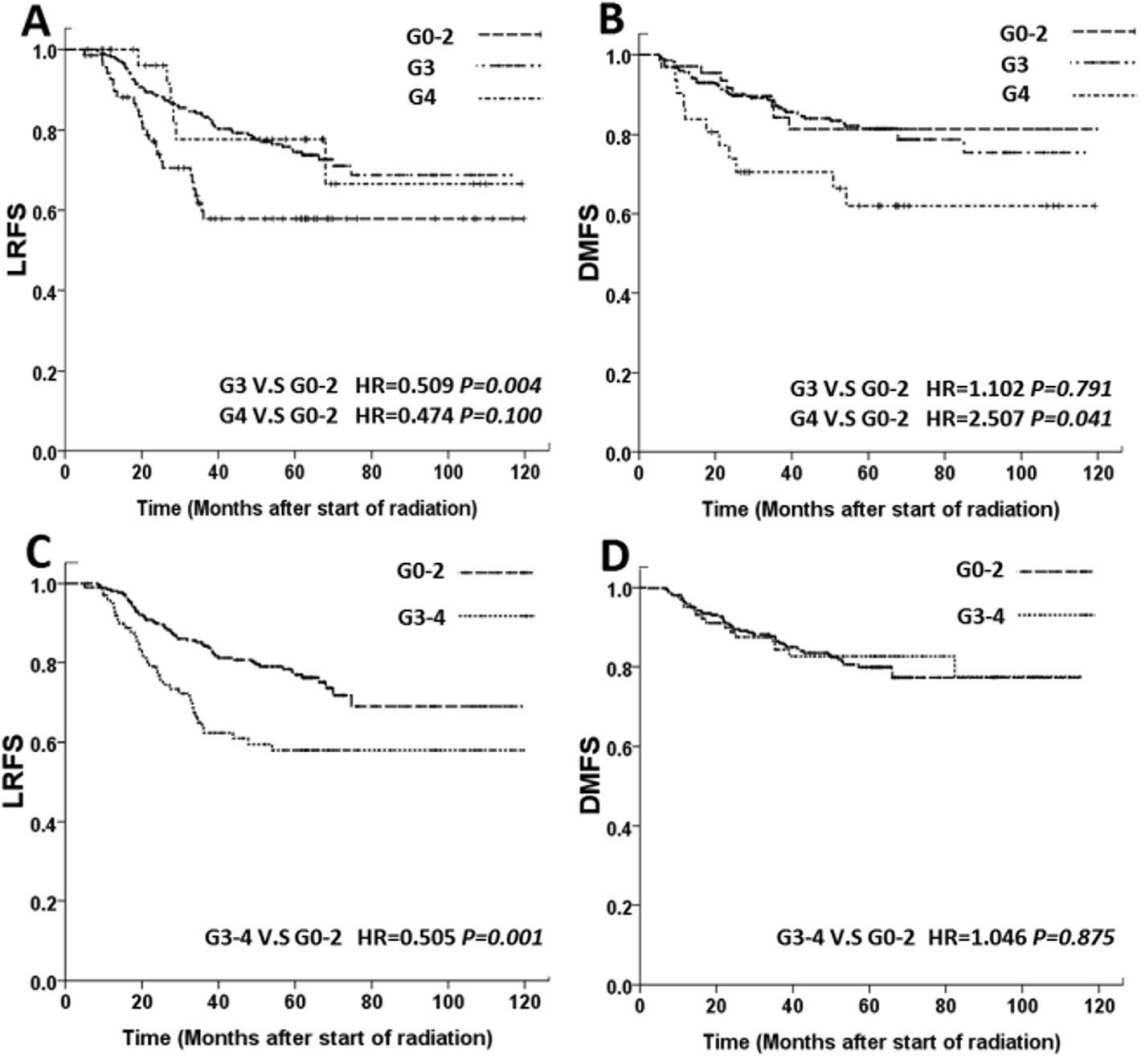
Kaplan-Meier curves show distant metastasis-free survival (DMFS) (**a** and **c**), local recurrence-free survival (LRFS) (**b** and **d**) of nasopharyngeal patients, grouped by lymphopenia grade (Common Terminology Criteria for Adverse Events 4.0) based on ALC nadir during RT

Of the 374 patients who were available for survival analysis, 158(42.25%) experienced events within the follow-up period, including 85(22.72%) with local regional failure, 65(17.38%) with distant metastatic failure, and 8(2.14%) with local regional and metastatic failure at the same time. The survival curves shown in Fig. 3 illustrate the correlations between ALC nadir and LRFS and DMFS. Log-rank tests and univariate analysis showed significant relationships between ALC nadirs and LRFS. Patients with G3–4 nadirs showed longer LRFS duration when compared to G0–2 nadir (Fig. 3a, c and Table 4); No significant difference was found between G3–4 nadir and G0–2 nadir for DMFS durations (Fig. 3d and Table 4). However, patients with G4 nadir during RT were at a higher risk of distant metastasis while comparing to G0–2 nadir during RT (Fig. 3b and Table 4).

**Table 4:** Univariate Cox regression analysis demonstrates associations between ALC nadirs and baseline variables and outcomes during RT

Characteristic	PFS			DMFS			LRFS		
	HR	95%CI	*P* value	HR	95%CI	*P* value	HR	95%CI	*P* value
RIL grade (nadir)									
G3 V.S G0–2	0.662	0.451–0.970	***0.034***	1.102	0.538–2.255	*0.791*	0.509	0.322–0.807	***0.004***
G4 V.S G0–2	0.998	0.559–1.781	*0.993*	2.507	1.038–6.053	***0.041***	0.474	0.195–1.153	*0.1*
G3–4 V.S G0–2	0.668	0.480–0.930	***0.017***	1.046	0.594–1.842	*0.875*	0.505	0.333–0.766	***0.001***
Sex									
Female	1			1			1		
Male	1.6	1.103–2.321	***0.013***	2.129	1.167–3.882	***0.014***	1.245	0.792–1.958	*0.342*
Age at diagnosis (years)	0.996	0.980–1.013	*0.649*	1.003	0.980–1.028	*0.78*	0.991	0.970–1.012	*0.388*
Lymphocyte baseline	0.992	0.740–1.331	*0.958*	0.928	0.585–1.472	*0.751*	1.039	0.710–1.521	*0.844*
Body mass index	0.975	0.927–1.025	*0.319*	0.992	0.920–1.065	0.779	0.956	0.893–1.023	*0.193*
Smoking status (pack*year)	1	1.000–1.001	***0.039***	1	1.000–1.001	*0.128*	1	1.000–1.001	*0.192*
Tumor histology (WHO)									
Undifferentiated (III)	1			1			1		
Differentiated (I + II)	1.402	1.003–1.960	***0.048***	1.445	0.880–2.371	*0.146*	1.285	0.836–1.973	*0.253*
Stage									
II	1			1			1		
III	1.335	0.786–2.266	*0.285*	1.811	0.715–4.587	*0.21*	1.257	0.662–2.385	*0.485*
IVa	1.78	1.001–3.163	***0.049***	3.41	1.307–8.897	***0.012***	1.095	0.517–2.321	*0.813*
Prescribed dose (Gy)	0.994	0.955–1.033	*0.749*	1	0.942–1.061	*0.991*	0.986	0.939–1.036	*0.575*
Induction or concurrent chemotherapy									
None	1			1					
Induction only	0.545	0.333–0.890	***0.015***	0.8	0.367–1.743	*0.574*	0.472	0.257–0.867	***0.016***
Concurrent only	0.463	0.272–0.790	***0.005***	0.85	0.385–1.876	*0.688*	0.286	0.136–0.601	***0.001***
Both	0.477	0.315–0.724	***< 0.001***	0.673	0.338–1.339	*0.259*	0.388	0.232–0.649	***< 0.001***
Adjuvant chemotherapy									
No	1			1			1		
Yes	0.959	0.687–1.338	*0.806*	1.341	0.837–2.150	*0.223*	0.69	0.435–1.094	*0.115*
Radiation modality									
2D-CRT	1			1			1		
IMRT	0.857	0.606–1.210	*0.38*	0.998	0.609–1.636	*0.994*	0.783	0.495–1.238	*0.295*

*Abbreviations*: *CI* confidence interval, *HR* hazard ratio, *2D-CRT* 2-dimensional conventional radiotherapy, *IMRT* intensity modulated radiation therapy, *WHO* World Health Organization

In the multivariate analysis, G3–4 nadir showed longer LRFS duration (vs. G0–2 nadir, HR =0.548, *P = 0.005*), and longer PFS duration (vs. G0–2 nadir, HR =0.676, *P = 0.022*); No significant difference was found between patients with G3–4 nadir and G0–2 nadir for DMFS. While patients with G4 nadir during RT showed shorter DMFS (vs. G0–2 nadir, HR =2.567, *P = 0.037*) during RT (Table 5).

**Table 5:** Multivariate Cox regression analysis demonstrates associations between ALC nadirs and baseline variables and outcomes during RT

Characteristic	PFS			DMFS			LRFS		
	HR	95% CI	*P* value	HR	95% CI	*P* value	HR	95% CI	*P* value
RIL grade (nadir)									
G3 V.S G0–2	0.637	0.434–0.936	***0.022***	NI	–	*–*	0.491	0.310–0.780	***0.003***
G4 V.S G0–2	NI	–	*–*	2.567	1.059–6.219	***0.037***	NI	–	*–*
G3–4V.S G0–2	0.676	0.484–0.945	***0.022***	NI	–	*–*	0.548	0.360–0.835	***0.005***
Sex									
Female	1			1			NI		
Male	1.556	1.070–2.263	***0.021***	2.005	1.097–4.080	***0.025***	NI	–	*–*
Stage									
II	1			NI			NI		
III	1.632	0.940–20,832	0.082	NI	–	*–*	NI	–	*–*
IVa	2.04	1.115–3.731	***0.007***	NI	–	*–*	NI	–	*–*
Induction or concurrent chemotherapy									
None	1						1		
Induction only	0.422	0.254–0.701	***0.001***	NI	–	*–*	0.386	0.210–0.709	***0.002***
Concurrent only	0.299	0.172–0.520	***< 0.001***	NI	–	*–*	0.206	0.098–0.434	***< 0.001***
Both	0.294	0.191–0.453	***< 0.001***	NI	–	*–*	0.265	0.159–0.443	***< 0.001***

*Abbreviations*: *HR* hazard ratio, *CI* confidence interval, *SD* standard deviation, *GTV* gross tumor volume, *ALC* absolute lymphocyte count

## Discussion

### RIL is affected by radiation therapy modality and baseline lymphocyte count

In this study, we evaluated the correlation between patient-, tumor-, and treatment-related features and RIL in NPC patients. We found that both baseline ALC and RT modality were independently associated with RIL.

Although only 33 (8.82%) patients were diagnosed with lymphopenia (ALC baseline < 1 × 10^9^ cells/L) before treatment, relatively lower level of lymphocyte baseline was associated with both incidence of G3 and G4 RIL, regardless of induction chemotherapy or concurrent chemotherapy use. Thus, the high-level lymphopenia during RT may have already been present (i.e., intrinsic immunosuppression that was enhanced by RT).

In this study, we showed that IMRT led to a greater decrease in ALC than did 2D-CRT. The analysis of RT techniques (IMRT vs. 2D-CRT) indicated that the incidence of high-level RIL during RT was significantly higher after IMRT than after 2D-CRT. Nevertheless, 2D-CRT is unlikely to be a better choice than IMRT after a comprehensive evaluation. Instead, the regular usage of low-dose volume restriction of surrounding normal tissue should be considered when planning IMRT. It is known that IMRT can greatly improve the uniformity and conformity of dose distribution on target volume compared with traditional 2D-CRT by compromising a larger volume of low-dose irradiation upon surrounding normal tissue [14]. Lymphocytes are known to be the most radiosensitive of the peripheral blood cells, with an LD_50_ as low as 2 Gy [15], and the incidental doses received when lymphocytes are within the radiation portal during fractionated RT could be sufficient to result in lymphopenia [16]. In reality, any low-dose-irradiated tissue surrounding the target volume can be considered the organ at risk for lymphopenia because peripheral lymphocytes circulate throughout the body and exist in all tissues. Thus, a large volume of even very low-dose irradiation (i.e., a greater “low-dose bath”) may result in more lymphocyte destruction. In order to compare the dose distribution between 2D-CRT and IMRT plans, we created 2D-CRT plans on 6 patients who had received IMRT. It showed that compared with IMRT the absolute total volume of receiving at least 5Gy (body V5) in 2D-CRT was greatly reduced (Supplementary fig. 1 and supplementary table 1). For the limitations of 2D-CRT, it is no longer commonly used for NPC patients. Therefore, the most important recommendation from our results should be restriction on low-dose irradiation(e.g. Body V5)in IMRT plans.

### ALC nadir during RT correlates with survival

In the survival analysis, Patients with G3–4 nadir during RT were at a lower risk of local reginal recurrence than patients with G0–2 lymphopenia (HR = 2.567, *P = 0.037*).

A large number of retrospective and prospective studies have shown that low lymphocyte nadir is associated with poor patient outcomes [17–23]. Cho et al. [12] reported that NPC patients with a minimum ALC < 245 cells/μL had worse PFS durations. Liu et al. [13] found that lymphopenia (mini-ALC < 390 cells/μL and post3m-ALC < 705 cells/μL) was strongly correlated with shorter DMFS and PFS durations in patients with NPC. Since a large number of publications have reported the correlation between lymphopenia and poor prognosis, we were surprised that G3–4 lymphopenia was beneficial predictor of LRFS and PFS in the present study.

Generally, RT can cause severe lymphopenia, leading to the suppression of host anti-cancer immunity; however, it can also stimulate tumor antigen release, which activates the T lymphocytes and enhances the host’s anti-cancer immunity. In fact, the presence of tumor-infiltrating lymphocytes, which are mainly composed of cytotoxic T lymphocytes, has been associated with better patient outcomes in melanoma and head and neck cancers [24, 25]. On the basis of our results, we assumed that, besides direct damage to lymphocytes by RT, lymphocytes infiltrating from the peripheral blood after stimulation with RT may lead to a reduction in circulating lymphocytes, which presents as lymphopenia. On the other hand, the radiosensitivity of lymphocytes may also be representative of the radiosensitivity of cancer cells [26], which predicts better survival.

However, among the 4 grades of lymphopenia, G4 nadir was associated with the shortest DMFS duration, which might be attributable to more immuno-suppression than immuno-stimulation induced by RT. Based on the result, it seems reasonable why no advance was found for PFS and OS in patients with G3–4, which showed advance for LRFS though. Therefore, G3 but not G4 nadir during RT may be indicative of an individually appropriate radiation dose that is able to stimulate anti-cancer immunity and defeat cancer cells without significantly influencing the host’s anti-cancer immunity.

Our study is subject to several limitations. In the survival analysis, we did not observe a significant correlation either between OS and RIL, or between OS and any patient-, tumor-, or treatment-related features (e.g., sex, age, clinical disease stage, radiation modality, and chemotherapy condition). We studied patients over 12 years; the substantial changes in social environments and common lifestyles could have contributed to the negative result in the OS analysis. In addition, our data were exclusively obtained from 1 center; the findings from the current study should be validated in a larger, multicenter study.

In conclusion, baseline ALC and RT modality, which were independently associated with RIL, should be considered carefully when developing a personalized RT plan. Moreover, G3–4 nadir was linked with longer LRFS durations, while G4 nadir was linked with a shorter DMFS duration; thus, routinely follow-up is more important for patients with G4 nadir as well as those with G0–2 nadir.

## Supplementary information

**Additional file 1: Table S1.** Comparison of V5 (mm^3^) between IMRT and 2D-CRT plans in 6 patients

**Additional file 2: Figure S1.** Dosimetric comparison between 2D-CRT (A, B, C) and IMRT (D, E, F). Note: The yellow line indicates the 5Gy isodose contour

## Data Availability

The datasets analyzed during the current study are available from the corresponding author on reasonable request.
