# Supplementary material for: Radiation-induced lymphopenia correlates with survival in nasopharyngeal carcinoma: impact of treatment modality and the baseline lymphocyte count
Source: Radiat Oncol. 2020 Mar 14;15:65. doi: 10.1186/s13014-020-01494-7 (PMC7071662; doi:10.1186/s13014-020-01494-7)
Supplement: Supplementary file 1 — Additional file 1: Table S1. Comparison of V5 (mm3) between IMRT and 2D-CRT plans in 6 patients [file 13014_2020_1494_MOESM1_ESM.docx]

Supplementary Table 1. Comparison of V5 (mm^3^) between IMRT and 2D-CRT plans in 6 patients.

| Patients | IMRT | 2D-CRT |
| --- | --- | --- |
| No.1 | 9729.68 | 6598.50 |
| No.2 | 6303.51 | 4395.13 |
| No.3 | 7683.72 | 5796.01 |
| No.4 | 6150.81 | 4358.14 |
| No.5 | 3852.99 | 2617.08 |
| No.6 | 4788.41 | 3392.88 |
